# Supplementary material for: Vitamin K2 sensitizes the efficacy of venetoclax in acute myeloid leukemia by targeting the NOXA-MCL-1 pathway
Source: PLoS One. 2024 Jul 25;19(7):e0307662. doi: 10.1371/journal.pone.0307662 (PMC11271855; doi:10.1371/journal.pone.0307662)
Supplement: S1 Fig — (PDF) [file pone.0307662.s001.pdf]

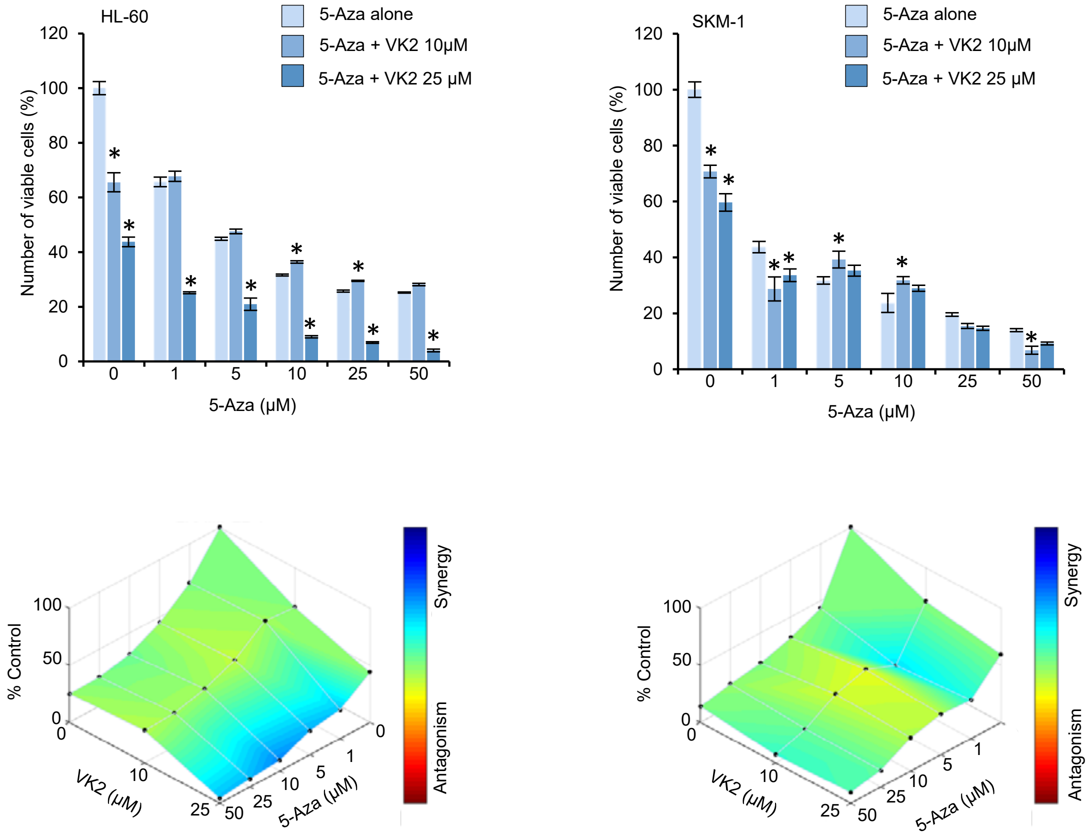

S1 Fig. Combination treatment of VK2 and AZA did not show synergistic cell death in AML cell lines. AML cell lines (HL-60 and SKM-1) were treated with 5-Aza in the presence or absence of VK2 at indicated concentrations for 48 h. Upper: The viable cell number was assessed using the CellTiter Blue assay. Data are presented as the mean ± SD. *$p<0.05$ vs. 0 μM VK2. Lower: The synergistic effect of combined VK2 and 5-Aza treatment on AML cell proliferation inhibition was statistically analyzed using the Combenefit software. Mapping of synergy levels on the experimental combination dose-response surface. A high score, shown in dense blue, indicates a strong synergistic effect. n=3
