# Supplementary material for: Vitamin K2 sensitizes the efficacy of venetoclax in acute myeloid leukemia by targeting the NOXA-MCL-1 pathway
Source: PLoS One. 2024 Jul 25;19(7):e0307662. doi: 10.1371/journal.pone.0307662 (PMC11271855; doi:10.1371/journal.pone.0307662)
Supplement: S2 Fig — (PDF) [file pone.0307662.s002.pdf]

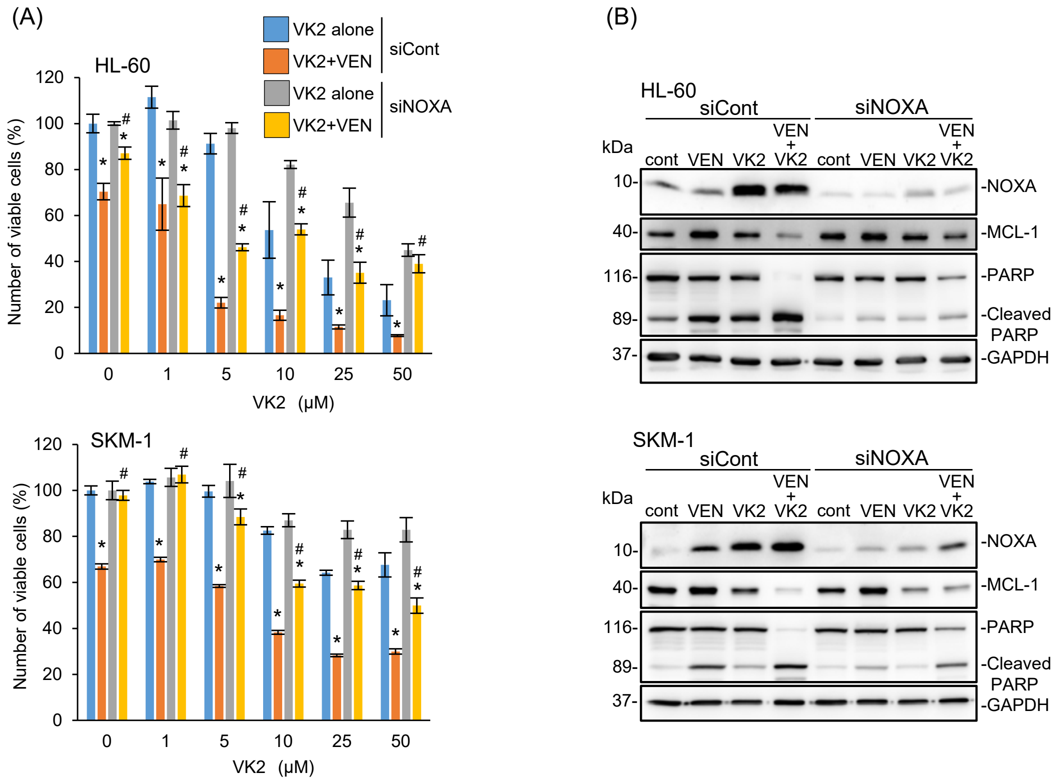

S2 Fig. *NOXA*-knockdown attenuated enhanced cell death caused by coadministration of VK2 and VEN. (A) HL-60 and SKM-1 cells were introduced control siRNA (siCont) or siRNA against *NOXA* (siNOXA) and then treated with VK2 and/or VEN (25 nM for HL-60, 2.5 μM for SKM-1) for 48 hr. The viable cell number was assessed by CellTiter Blue assay. Data are presented as the mean ± SD. *P<0.05 vs. VEN 0 nM, #P<0.05 v.s. siCont. (B) HL-60 and SKM-1 cells were introduced siCont or siNOXA and then treated with VK2 (10 μM for HL-60, 25μM for SKM-1) and/or VEN (25 nM for HL-60, 2.5 μM for SKM-1) for 48 hr. Cellular proteins were lysed, separated by SDS-PAGE, and immunoblotting was performed using indicated antibodies.
