# Supplementary material for: Vitamin K2 sensitizes the efficacy of venetoclax in acute myeloid leukemia by targeting the NOXA-MCL-1 pathway
Source: PLoS One. 2024 Jul 25;19(7):e0307662. doi: 10.1371/journal.pone.0307662 (PMC11271855; doi:10.1371/journal.pone.0307662)
Supplement: S3 Fig — (PDF) [file pone.0307662.s003.pdf]

Fig.2 (E)

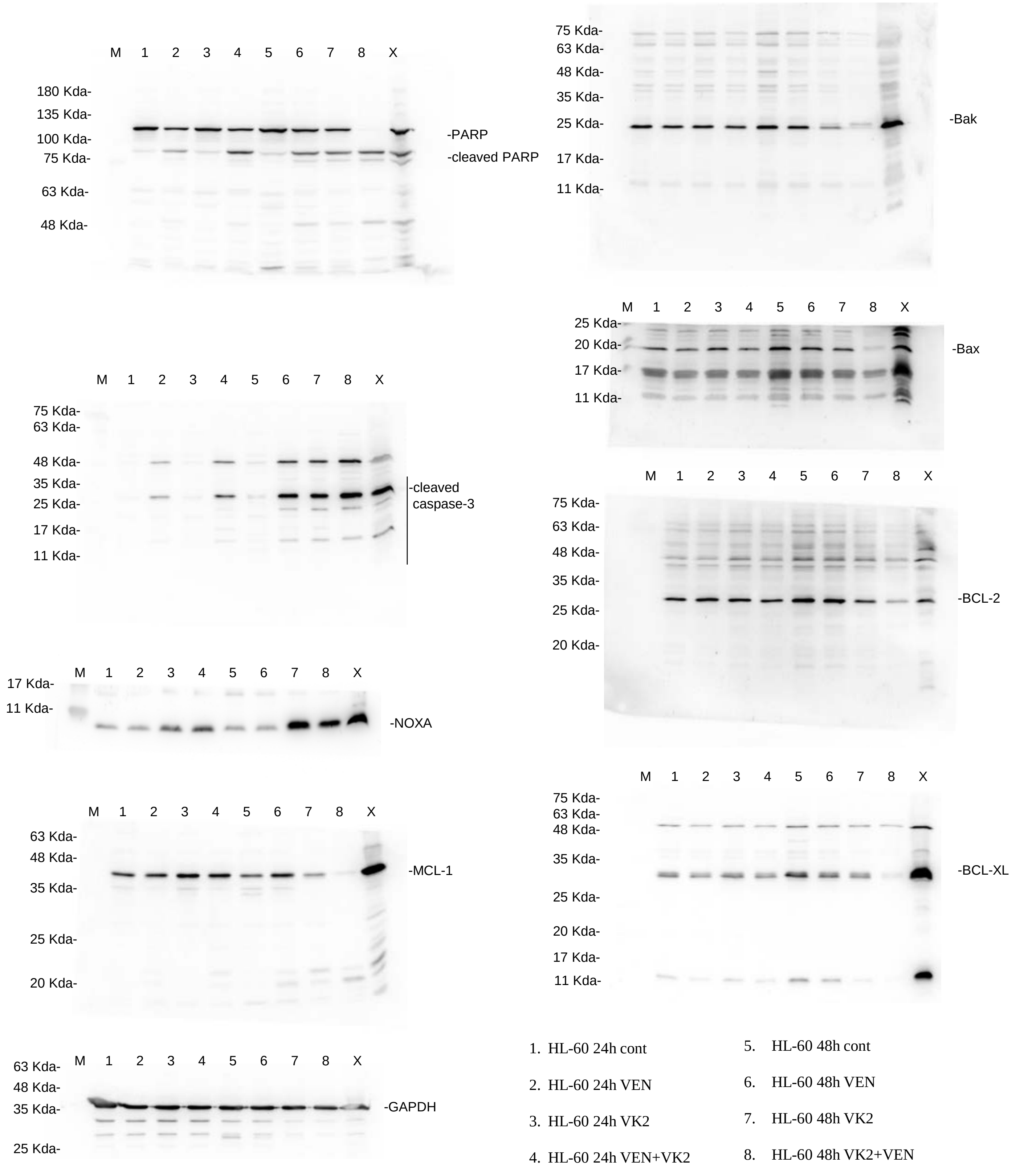

1. HL-60 24h cont
2. HL-60 24h VEN
3. HL-60 24h VK2
4. HL-60 24h VEN+VK2
5. HL-60 48h cont
6. HL-60 48h VEN
7. HL-60 48h VK2
8. HL-60 48h VK2+VEN

Fig.2 (F)

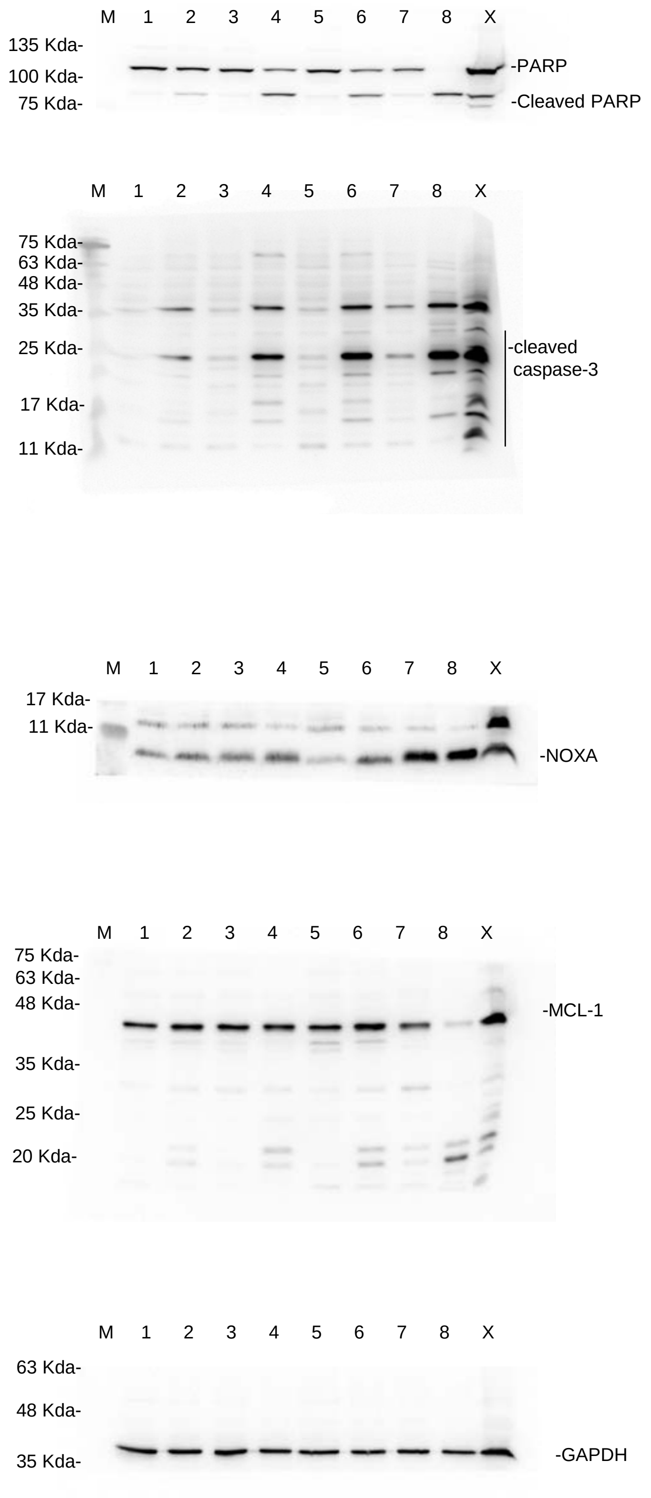

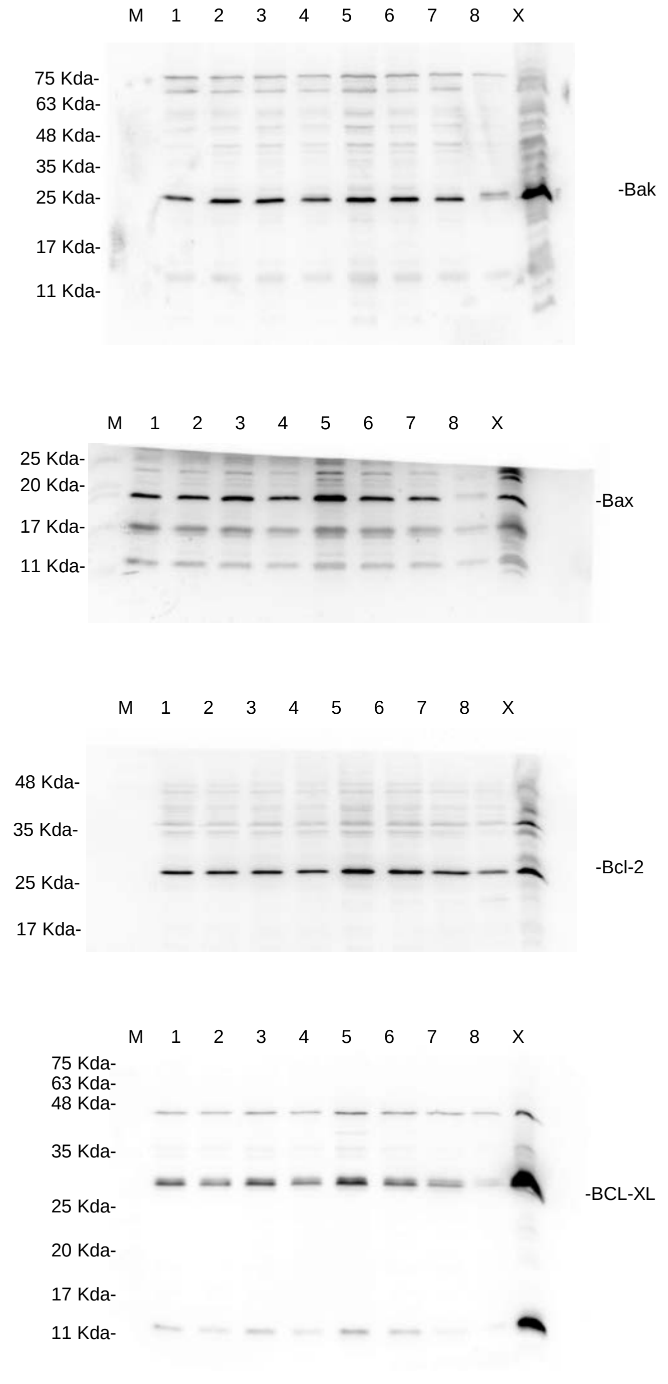

1. SKM-1 24h cont
2. SKM-1 24h VEN
3. SKM-1 24h VK2
4. SKM-1 24h VEN+VK2
5. SKM-1 48h cont
6. SKM-1 48h VEN
7. SKM-1 48h VK2
8. SKM-1 48h VK2+VEN

Fig.4 (I)

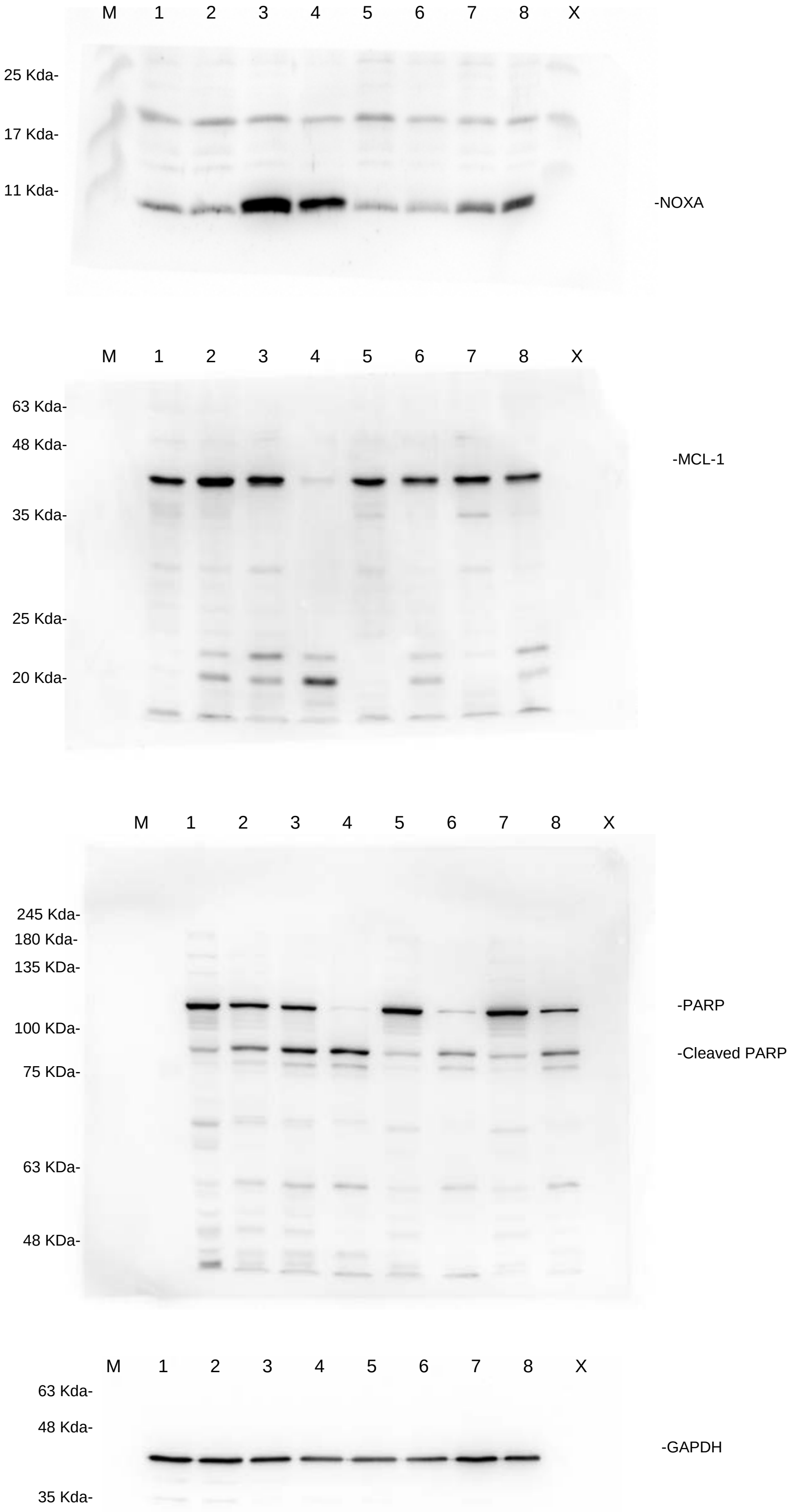

1. HL-60 NAC (-) cont
2. HL-60 NAC (-) VEN
3. HL-60 NAC (-) VK2
4. HL-60 NAC (-) VEN+VK2
5. HL-60 NAC (+) cont
6. HL-60 NAC (+) VEN
7. HL-60 NAC (+) VK2
8. HL-60 NAC (+) VK2+VEN

Fig.4 (J)

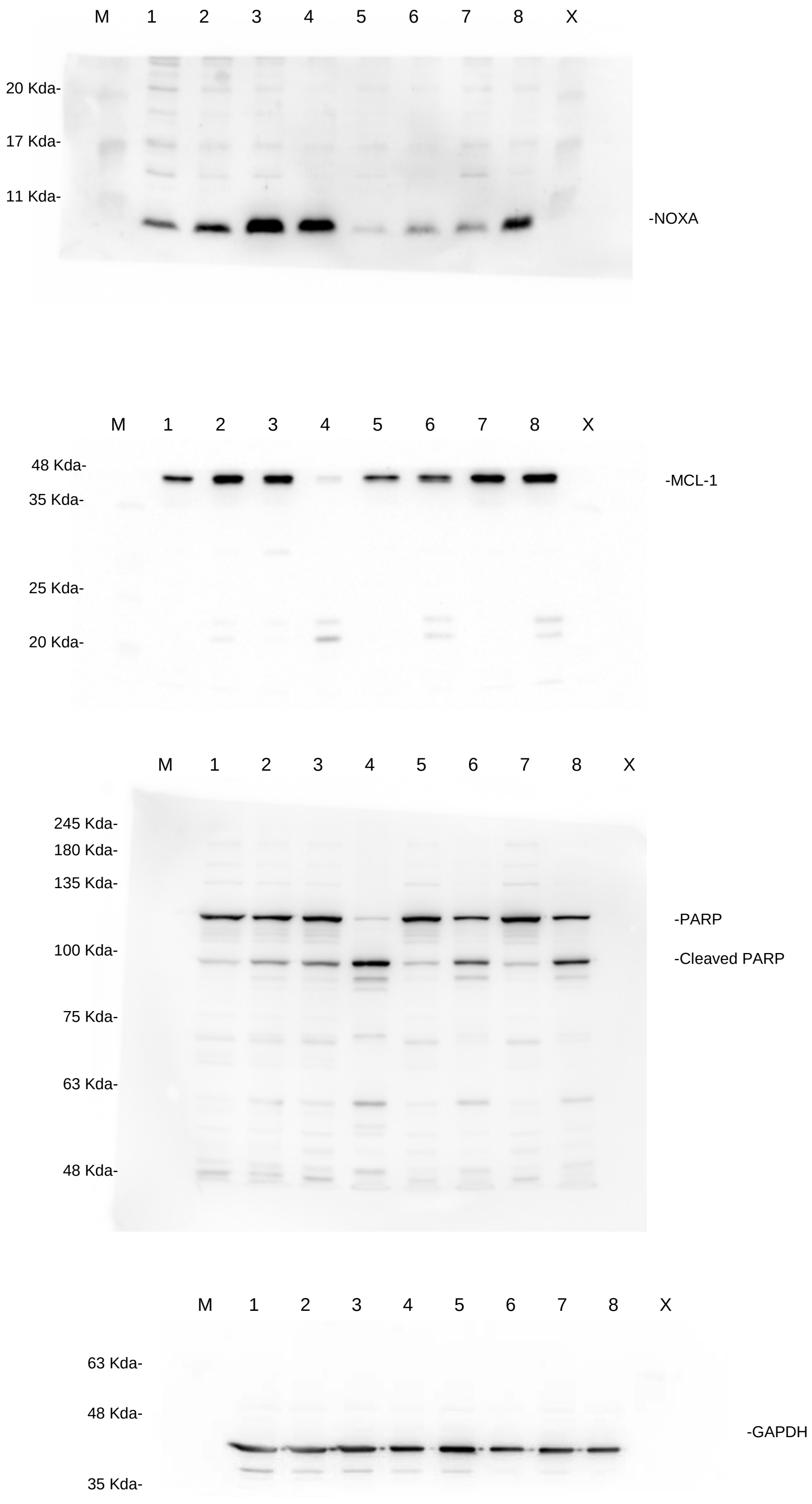

1. SKM-1 NAC (-) cont
2. SKM-1 NAC (-) VEN
3. SKM-1 NAC (-) VK2
4. SKM-1 NAC (-) VEN+VK2
5. SKM-1 NAC (+) cont
6. SKM-1 NAC (+) VEN
7. SKM-1 NAC (+) VK2
8. SKM-1 NAC (+) VK2+VEN

Fig.S2 / HL-60

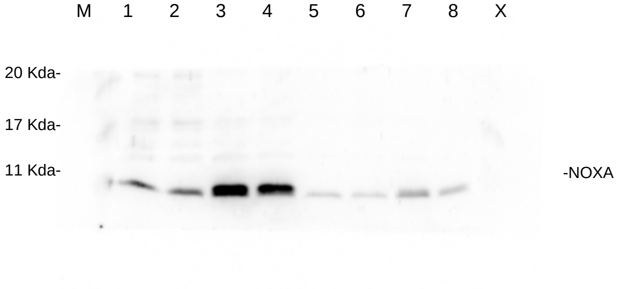

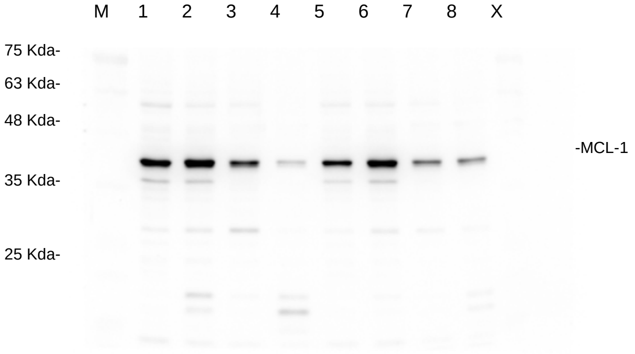

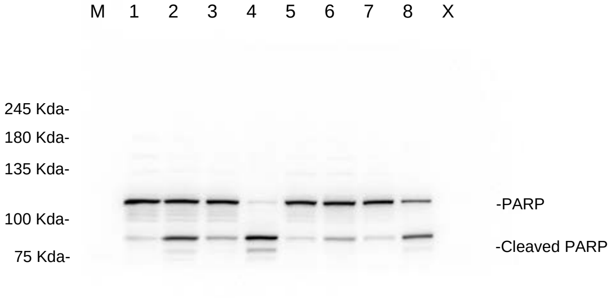

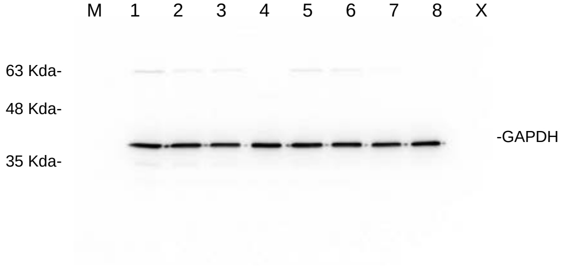

1. HL-60 siCont cont
2. HL-60 siCont VEN
3. HL-60 siCont VK2
4. HL-60 siCont VEN+VK2
5. HL-60 siNOXAcont
6. HL-60 siNOXAVEN
7. HL-60 siNOXAVK2
8. HL-60 siNOXAVK2+VEN

Fig.S2 / SKM-1

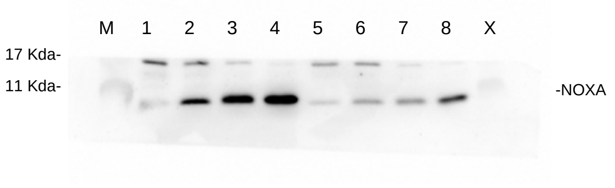

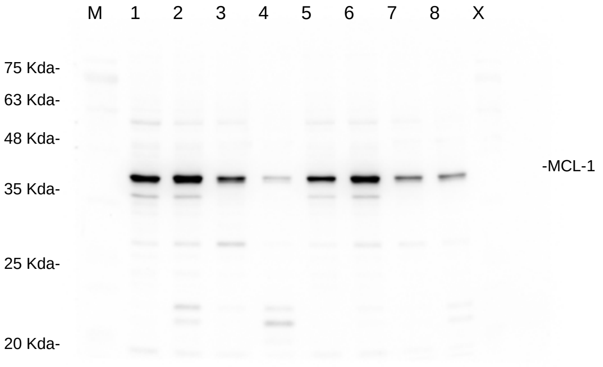

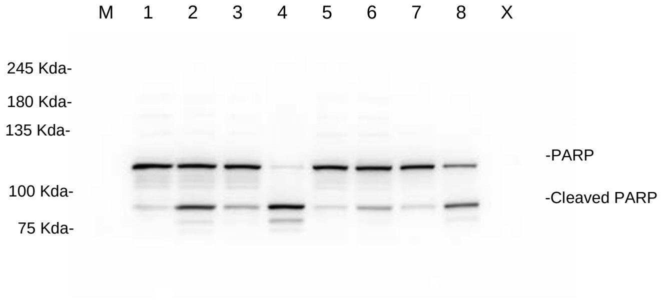

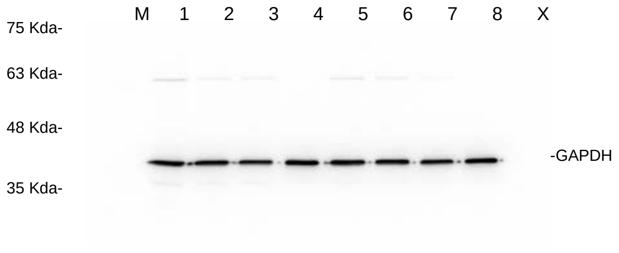

1. SKM-1 siCont cont
2. SKM-1 siCont VEN
3. SKM-1 siCont VK2
4. SKM-1 siCont VEN+VK2
5. SKM-1 siNOXAcont
6. SKM-1 siNOXAVEN
7. SKM-1 siNOXAVK2
8. SKM-1 siNOXAVK2+VEN
